# A New LBP Variant: Corner Rhombus Shape LBP (CRSLBP)

**DOI:** 10.3390/jimaging8070200

**Published:** 2022-07-17

**Authors:** Ibtissam Al Saidi, Mohammed Rziza, Johan Debayle

**Affiliations:** 1 LRIT Laboratory, Rabat IT Center, Faculty of Sciences, Mohammed V University in Rabat, Rabat B.P. 1014, Morocco; mohammed.rziza@gmail.com; 2 Mines Saint-Etienne, French National Center for Scientific Research, Joint Research Unit 5307 Laboratory Georges Friedel, Centre SPIN 158 Cours Fauriel, CEDEX 2, 42023 Saint-Etienne, France; debayle@emse.fr

**Keywords:** feature extraction, local binary pattern, texture classification

## Abstract

The local binary model is a straightforward, dependable, and effective method for extracting relevant local information from images. However, because it only uses sign information in the local region, the local binary pattern (LBP) is ineffective at capturing discriminating characteristics. Furthermore, most LBP variants select a region with one specific center pixel to fill all neighborhoods. In this paper, a new variant of a LBP is proposed for texture classification, known as corner rhombus-shape LBP (CRSLBP). In the CRSLBP approach, we first use three methods to threshold the pixel’s neighbors and center to obtain four center pixels by using sign and magnitude information with respect to a chosen region of an even block. This helps determine not just the relationship between neighbors and the pixel center but also between the center and the neighbor pixels of neighborhood center pixels. We evaluated the performance of our descriptors using four challenging texture databases: Outex (TC10,TC12), Brodatz, KTH-TIPSb2, and UMD. Various extensive experiments were performed that demonstrated the effectiveness and robustness of our descriptor in comparison with the available state of the art (SOTA).

## 1. Introduction

Texture, as a significant characteristic, can be depicted as part of the large ambit of an object surface or set of object images, comprising size, illumination, organization, color, and other physical or natural features. In 2002, a new statistical method for image processing and pattern recognition was proposed by [1,2], which was the first to introduce local binary patterns (LBPs). The main purpose of this new method is to process a textured image using a particular kernel function that constitutes the statistical relationship between neighbors and the center and allows one to compute the transformation value by capturing local structural patterns. The simplicity, robustness, and rapidity of the LBP calculation has attracted attention from researchers looking to create their own local operators by developing other variants.

The authors of [3] remarked that the high frequency of occurrences counted by an LBP could achieve predominant texture information by introducing dominant local binary patterns (DLBPs). The original LBP method was extended by [4] to solve noise limitation by using three rather than two valued codes, which they called a local ternary pattern (LTP). Guo et al. [5] introduced completed LBP (CLBP) modeling, which took into consideration both magnitude (CLBP-magnitude) and sign (CLBP-sign). Furthermore, the CLBP-center contained the same information as an LBP. To overcome the high sensitivity to noise of CLBP and dimensionality, Liu et al. [6] proposed binary rotation invariant and noise tolerant (BRINT) texture classification, which combines three descriptors—BRINT S, BRINT M, and BRINT C—which enhance noise tolerance by quantizing the average gray pixel value. A scale-selective LBP (SSLBP) was suggested by [7] to take the pre-learned dominant LBP pattern at variant scale spaces. To maintain good discriminant features, Liu et al. [8] proposed a median robust Extended LBP (MRELBP) that uses regional image medians rather than raw image intensities. This method combines three descriptors: MRELBP NI, MRELBP RD, and MRELBP CI. A radial mean LBP (RMLBP) was suggested by [9] to solve the problem of noise sensitivity by using the mean of points over each radial instead of employing angular neighbor points. In [10] a cross-complementary LBP (CCLBP) was proposed to enhance the robustness to scale, viewpoint, and number of training samples by diversifying two parameters accordingly. Recently, many other interesting modifications and improvements to LBPs have been developed: LOOP [11], ACS-LBP and RCS-LBP [12], MLD-CBP [13], CLSP [14], LCvMSP [15], Hess-ACS-LBP [16], ACPS [17], and LDT [18].

In texture classification, many descriptors and extensions of an LBP use just one center as a reference to threshold the neighboring pixels. Therefore, the relationship between the center pixels is loosened. Furthermore, a LBP uses bilinear interpolation, which has many limitations such as the loss of sharpness, inaccuracy of the gray value, and high computational complexity. A new LBP version is proposed in this article to overcome these weaknesses: the corner rhombus-shaped LBP (CRSLBP). In fact, the CRSLBP is an improved version of the LBP method because it takes into consideration sign and magnitude information and uses a single parameter (radius) with the addition of the chosen even block, which permits the thresholding of four center pixels. This serves to obtain relationships not only between neighbors, but also between the centers and the neighbor of centers. Three different processes are used to obtain three descriptors that give a better characterization of images. The histogram of each image is extracted and concatenated with the others to obtain discriminant and robust features. Specifically, to obtain more than one center, the CRSLBP uses 4 × 4 blocks to select four center pixels at the same time. From this, the relationship between the center pixels and between the center and neighbors of the neighboring center pixels can be determined. Furthermore, bilinear interpolation is eliminated, so all focus is on information from the block and exploiting it using various thresholding methods that have been adaptively computed by examining local structures and their properties.

This study is structured as follows: Section 2 and Section 3 introduce a brief related work and the proposed texture analysis descriptor: the corner rhombus-shaped LBP (CRSLBP), respectively. Section 4 discuses the performance of the proposed method by using classifiers compared to SOTA approaches. The paper is concluded in Section 5.

## 2. Related Work

Before going into our proposed approach, we first need to present a brief review of the main works in the literature that inspired us. We start with the original LBP and then present the motivation that gave us the idea for our new method.

### 2.1. Local Binary Pattern (LBP)

The original LBP was created by [1,2] with 3 × 3 blocks containing eight neighbors with a center to capture important local information. The LBP code feature is generated by the following equation:

(1) $$LBP_{R,P}\left(c\right)=\sum_{i=0}^{P-1}s\left(g_{i}-g_{c}\right)2^{i},s\left(x\right)=\left\{\begin{array}{cc} 1 & x\geq 0\\ 0 & otherwise \end{array},\right.$$

where $g_{c}$ and $g_{i}$ represent, respectively, the center pixel and its neighbors on the *i*-th position with radius *R*; *P* is the number of samples; and s() is the sign function.

### 2.2. Research Motivation

By recovering the publications of LBP variants from this year, we discovered that most approaches used one center as a reference to threshold all neighbors and replaced it with LBP code. Consequently, the relationship between the centers is loosened. On the other hand, the bilinear interpolation that the LBP used made possible the calculation of the value, which is supposed to be placed at the same distance from the central pixel (gray circle). However, it has many weaknesses such as the loss of sharpness, inaccuracy of the gray value, imprecise texture information, and high computational complexity.

To avoid these issues and limitations, we created a new LBP variant with big differences in the form, shape, and code of the extracted local pattern. The problems were solved by mapping the code LBP with even blocks, as opposed to most LBP variants that use odd blocks to select one center with their neighbors. In this way, we had the chance to work with four centers at the same time, allowing us not only to obtain the interconnection between the center pixels, but also each center pixel with its neighbors. Furthermore, each center pixel gained a relationship with the neighbor pixels of the center neighborhood pixels. Additionally, the new proposed descriptor eliminated bilinear interpolation and exploited all the information provided by the neighboring pixels in the block using multiple thresholds computed adaptively by examining different local structures and their properties.

On the other hand, we extracted information from the relationship between neighbors based on the center pixels. As provided in CLBP, and to preserve more intrinsic features, two important vectors were extracted from the image: sign and magnitude. However, of the two, the sign was the most influential. Based on this idea, we extracted the sign from the rib pixels of the rhombus-shaped neighbor pixels. In addition, to obtain a depth relationship between the center and its neighbors, each pixel center was thresholded with the neighbors of the neighboring center.

Based on the preceding, this new encoding was useful for acquiring more intrinsic information, which allowed for a significant improvement in classification accuracy.

## 3. Proposed Methodology

**Figure 1:**
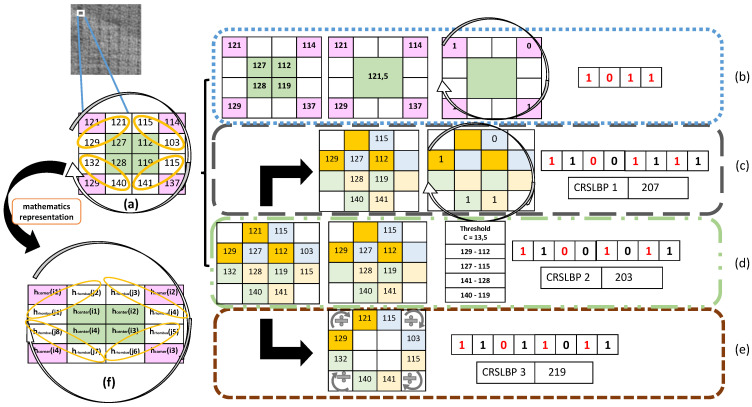
(**a**) The 4 × 4 sub-block of the image. (**b**) The corner processing. The process of the first (**c**), second (**d**), and third (**e**) generated CRSLBP code. (**f**) mathematical representation of the block.

In this section, we present our new LBP variant for texture classification to solve the weaknesses of the original LBP and to obtain more robust features with low complexity. In general, the CRSLBP is constructed in the following major steps. Contrary to the LBP and most of its variants, our input data were divided into even blocks of 4 × 4 pixels, making it possible to select four center pixels in each block $h_{center}\left(i\right)$ (see Figure 1f, the green one), and to exploit the relationship between the four centers and their neighbor pixels. These were partitioned into corner $h_{corner}\left(i\right)$ and rhombus-shaped neighbor pixels $h_{rhombus}\left(j\right)$ and are marked by pink and orange circles, respectively, in Figure 1.

After extracting all the required pixels, we began the construction of the binary encoded pattern as follows:

**Step 1:** The four selected corner pixels were compared by the mean of all center pixels, which gave four binary patterns (Figure 1b). (1) The corner neighbor pixels of the block $h_{corner}\left(i\right)$ were given by the following equation:

(2) $$\begin{array}{c} CRSLBP_{corner}^{riu2}\left(r,N\right)=\sum_{i=0}^{N-1}s\left(h_{corner}\left(i\right)-h_{Mcenter}\right),s\left(x\right)=\left\{\begin{array}{cc} 1 & x\geq 0\\ 0 & x<0 \end{array}\right. \end{array}$$

where *r* represent the radius and in our proposed method radius {1,2,3} is respectively the block of {(4×4), (6×6), (8×8)}; $h_{Mcenter}$ represent the mean of all centers pixels; and s() is the sign function.

**Step 2:** As shown in (Figure 1c) each specific rib of the rhombus contains two pixels. First, we took the maximum of the two pixels and compared it with the horizontal switching of the center pixels. This gave us four new binary patterns. Formally, the first process of the rhombus-shaped neighbor pixels is defined as:

(3) $$\begin{array}{c} CRSLBP_{rhombus1}^{riu2}\left(r,N\right)=\sum_{j=0}^{(N*2)-1}s\left(\max\left(h_{rhombus}\left(2j+1\right),h_{rhombus}\left(2j+2\right)\right)-h_{center}\right),\\ s\left(x\right)=\left\{\begin{array}{cc} 1 & x\geq 0\\ 0 & x<0 \end{array}\right. \end{array}$$

**Step 3:** We extracted the minimum and maximum numbers from each specific rib of the rhombus pixels and compared them with the horizontal switching of the center pixels, which created a relationship between each center and its far neighbors. Next, we calculated the C value, which is used to threshold the neighbors by subtracting the mean of all maximum numbers with the average of all the minimum numbers. We then subtracted the maximum numbers of each specific rhombus rib from the horizontal switching of the center pixels and compared them with the *C* value to generate another four binary-encoded patterns (Figure 1d). The second process of the rhombus-shaped neighbor pixels is given by:

(4) $$\begin{array}{c} CRSLBP_{rhombus2}^{riu2}\left(r,N\right)=\sum_{j=0}^{(N*2)-1}B\left(\max\left(h_{rhombus}\left(2j+1\right),h_{rhombus}\left(2j+2\right)\right)-h_{center}\right),\\ B\left(x\right)=\left\{\begin{array}{cc} 1 & x\geq C\\ 0 & x<C \end{array}\right. \end{array}$$

where B(x) is the sign function based on the contrast value. The *C* value, which is used to improve the quality of the image based on operations such as contrast enhancement and the reduction or removal of noise is calculated as follows:

(5) $$\begin{array}{c} C=1/N(\sum_{j=0}^{(N*2)-1}\max\left(h_{rhombus}\left(2j+1\right),h_{rhombus}\left(2j+2\right)\right)\\ \;\;\;\;\;\;\;\;\;\;\;\;\;\;-\sum_{j=0}^{(N*2)-1}\min\left(h_{rhombus}\left(2j+1\right),h_{rhombus}\left(2j+2\right)\right)). \end{array}$$

**Step 4:** For each specific rib of rhombus pixels following a particular direction as presented in (Figure 1e) the ratio of every two pixels was calculated, and the entire value was captured to extract four additional binary patterns. The last equation is defined as follows:

(6) $$\begin{array}{c} CRSLBP_{rhombus3}^{riu2}\left(r,N\right)=\sum_{j=0}^{(N*2)-1}s(h_{rhombus}\left(2j+1\right)/h_{rhombus}\left(2j+2\right)),\\ \;\;\;\;\;\;\;\;\;\;\;\;\;\;\;\;\;\;\;\;\;\;\;\;s\left(x\right)=\left\{\begin{array}{cc} 1 & x\geq 1\\ 0 & x<1 \end{array}\right. \end{array}$$

In Equations (3) and (4), the center $h_{center}$ (presented in Figure 1f) for thresholding each of rib rhombus shape neighbor pixel is organized as follows:

$h_{center}=\left\{h_{center2},h_{center1},h_{center4},h_{center3}\right\}$.

**Step 5:** Equations (2–4) and (6) generated four binary patterns. After extracting all of them, we formed three decimal codes by concatenating two four-binary patterns pixel by pixel, as follows:

(1) Step 1 with Step 2
  (7) $$CRSLBP1^{riu2}\left(r,N\right)=\sum_{i=0}^{\left(N-1\right)}\sum_{j=2i+1}^{\left(N-1\right)}\left(CRSLBP_{corner}^{riu2}\left(i\right)2^{\left(j-1\right)}+CRSLBP_{rhombus1(r,N)}^{riu2}\left(i\right)\right)2^{j}$$
(2) Step 1 with Step 3
  (8) $$CRSLBP2^{riu2}\left(r,N\right)=\sum_{i=0}^{\left(N-1\right)}\sum_{j=2i+1}^{\left(N-1\right)}\left(CRSLBP_{corner}^{riu2}\left(i\right)2^{\left(j-1\right)}+CRSLBP_{rhombus2}^{riu2}\left(i\right)\right)2^{j}$$
(3) Step 1 with Step 4
  (9) $$CRSLBP3^{riu2}\left(r,N\right)=\sum_{i=0}^{\left(N-1\right)}\sum_{j=2i+1}^{\left(N-1\right)}\left(CRSLBP_{corner}^{riu2}\left(i\right)2^{\left(j-1\right)}+CRSLBP_{rhombus3}^{riu2}\left(i\right)\right)2^{j}$$

The total process of the CRSLBP explained above is illustrated in Figure 1.

To increase the discrimination and effectiveness of the feature representation, the three encoded pattern processes CRSLBP 1–3, given in Equations (7)–(9) are grouped into a hybrid distribution named CRSLBP Equation (10), which allowed us to create a robust model with reduced noise sensitivity and improved effectiveness. In addition, by using a linear combination of several characteristics generated from different processes of pattern encoding, a multi-scale approach was used to capture coarse and fine information. The CRSLBP is presented as follows:

(10) $$CRSLBP^{riu2}\left(r,N\right)=\langle CRSLBP1^{riu2}\left(r,N\right),CRSLBP2^{riu2}\left(r,N\right),CRSLBP3^{riu2}\left(r,N\right)\rangle$$

**Figure 2:**
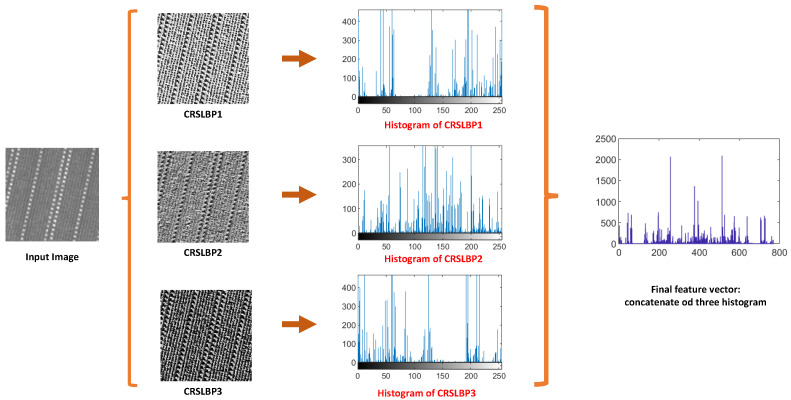
The concatenation histogram of all CRSLBP processes.

Figure 2 shows the texture features after CRSLBP extraction.

## 4. Experiment Results

This section concerns a series of experiments with various databases conducted to verify the effectiveness of the CRSLBP strategy.

### 4.1. Texture Datasets

Datasets from the Outex [19], KTH-TIPS2b [20], UMD [21] and Brodatz [22] representation databases were used in our experiments to evaluate the robustness and effectiveness of the proposed CRSLBP. Table 1 summarizes the information from each database. The suggested method is compared with other LBP variants, some of which are classified in the same category, “combining with complementary features”, as our method.

In the experiments in this paper, all descriptors were considered as parameters setting the rotation invariant and uniform (riu2) with normalized features to decrease the number of features, thereby reducing processing time and providing discriminating features. The suggested method was tested using a support vector machine (SVM) and neural network (NN) and compared to other LBP variants, some of which are classified in the same category, “combining with complementary features”, as our method. For a comparative result, the SVM classifier was trained with a radial basis function (RBF) kernel, which is one of the most widely used due to its similarity to the Gaussian distribution. The RBF kernel support vector machine depends highly on two hyperparameters: *C* for SVM and γ for the RBF Kernel, whereas the optimum value of *C* and gamma (γ) had been selected by the grid search method using 10-fold cross-validation.

### 4.2. Experimental Results of Outex Database

The classification results of this experiment are illustrated in Table 2.

First, we compared our descriptor with the original LBP method. Remarkably, the performance of the CRSLBP was much higher for all resolutions: various Outex (TC10 and TC12), the classifiers (SVM, NN), and the radius. The average classification accuracy was 99.76 and 99.79% for Outex TC10 SVM and NN, respectively. Additionally, we compared the CRSLBP with a homogeneous LBP (HLBP), homogeneous rotated LBP (HRLBP) and circular part LBP (CPLBP), which were introduced by [23,24,25]. As can be seen, our proposed method improved upon the HLBP, HRLBP and CPLBP descriptors, with higher classification accuracies in various illuminations of Outex (Inca, T184 and horizon) and for each proposed resolution (classifier, Outex databases, radius and homogeneity tolerance). Furthermore, we achieved the best results even though HLBP+LBP and HRLBP+RLBP were concatenated, demonstrating the robustness and high performance of our method: first, from the four center pixels extracted from the block; second, from the relationship derived from each center and neighbor of center pixels. Last, we compared our approach with SOTA approaches. As shown in the table, the average performance of the CRSLBP with both SVM and NN classifiers for all Outex types (TC10, TC12) was higher than the SOTA, apart from MRELBP [8]. It is normal to obtain small differences in classification accuracies between the two approaches (MRELBP and CRSLBP) owing to the set of four radius values used in MRELBP to generate a code that enables multiple scales at the same time.

### 4.3. Experimental Results with the KTH-TIPS2b Database

The KTH-TIPS2b [20] database is primarily designed to assess the impact of real-world imaging conditions on material classification. Table 3 displays its classification accuracy in evaluating the performance of our descriptor using SVM and NN classifiers

It can be seen that the CRSLBP outperformed the original LBP with various radius values and classifiers by over 8.87% for SVM and 10% for NN. Similarly, the CRSLBP had an average classification accuracy 5–10% higher than those presented by [23,24] for HLBP and HRLBP and their concatenation with LBP and RLBP, respectively. To further evaluate the performance of the CRSLBP, we made another comparison with some SOTA methods, as shown in the table. The CRSLBP achieved much better classification accuracies than LBP: 96.89, 96.76, and 97.19% for the SVM classifier and 94.81, 95.37, and 95.65% for NN classifier (radius R = 1, R = 2, and R = 3, respectively). Just like the Outex database, CRSLBP did not obtain better results on KTH-TIPS2b compared to the MRELBP.

### 4.4. Experimental Results with the UMD Database

The experimental results with the UMD dataset [21] are listed in Table 3 for both the SVM and NN classifiers. We primarily examined the CRSLBP in comparison with the original LBP. Despite the high resolution, arbitrary rotation, large changes in viewpoint. and different scales within the UMD dataset, the proposed approach obtained the highest accuracy: 100% for R = 2 with NN. For both classifiers, the CRSLBP was much more robust than the LBP. Our second experiment, tested it in comparison to HLBP and HRLBP. and CRSLBP displayed higher classification accuracy: 98.5, 98.4, and 98.80% with the SVM classier and 99.33, 100, and 98.67% with NN (radius R = 1, R = 2, and R = 3, respectively). Moreover, the CRSLBP demonstrated its robustness in comparison to the HLBP reinforced by the LBP and HRLBP reinforced by the RLBP. The last experiment showed the potential of the CRSLBP in comparison to SOTA approaches. As we can see, the average classification accuracy of our descriptor was much higher than the others across different resolutions (radius and classifier), except for the MRELBP, as explained before.

### 4.5. Experimental Results with Brodatz Database

The Brodatz [22] database, despite being relatively old, is still widely used. The experiment results with the Brodatz dataset are presented in Table 3. Using the CRSLBP, we obtained a 95.04 and 98.01% average classification accuracy, outperforming the original LBP, which had a 91.40 and 89.40% accuracy with SVM and NN, respectively, demonstrating the performance of the CRSLBP. On the other hand, we evaluated the robustness of the CRSLBP in comparison with the HLBP, HRLBP, HLBP+LBP and HRLBP+HRLBP, As shown in the tables, the highest accuracies were achieved by the CRSLBP, and SOTA methods, which yielded classification accuracies lower than ours with the exception of the MRELBP.

### 4.6. Experimental Results of CRSLBP with MRELBP

Concerning the low classification accuracy of our descriptor compared to the MRELBP method, it was normal to obtain small differences in classification accuracy between the two approaches owing to the set of four radius values used in the MRELBP to generate a code to enable multiple scales at the same time. To avoid this illegality between CRSLBP and MRELBP, we performed another experiment with lawful parameters. For both descriptors, the select parameters were a set of radius values (2, 4, 6, 8) with an SVM classifier. Table 4 shows the results of this experiment.

Based on the analysis of the table, our method performed better than the MRELBP, with higher classification accuracies for most of the datasets (Outex TC12 and KTH-TIPSb2). Furthermore, the differences in results with the other databases is very small if we look at the large difference in dimension between the two: 120 (30 × 4) and 800 for the CRSLBP and MRELBP.

## 5. Conclusions

This work proposed a new approach for texture classification images: “corner rhombus-shaped LBP” (CRSLBP). In fact, it is an improved version of the LBP method that took into consideration sign and magnitude with the addition of the chosen even block, which allowed us to threshold four center pixels. In this way, we obtained relationships not only between neighbors, but also between the center. A variety of challenging texture databases (Outex [TC10, TC12], Brodatz, UMD, and KTH-TIPSb2) and two classifier approaches (SVM and NN) were used to evaluate the proposed method.

The experimental results showed that the CRSLBP outperformed the LBP and its new variants: the HLBP, HRLBP, HLBP + LBP, HRLBP + RLBP and CPLBP. On the other hand, we evaluated the CRSLBP with other SOTA methods, and generally the experimental results show that the CRSLBP largely outperformed these methods in classification accuracy against various classification challenges, including strong scale, changes in rotation, scale, illumination, and viewpoint. However, the CRSLBP was expected to be robust for noise data using a variety of noises, which should be investigated in future work.

Our future work will consist also of testing our method with color image databases. However, this operator tends to produce high-dimensional feature vectors. Thus, to address this problem, we will focus on the application of feature selection methods to CRSLBP-based features. Another upcoming project will also include analyzing and comparing the many ways presented in the literature for exploiting the features of several color spaces at the same time.

## Figures and Tables

**Table 1:** Summary of the characteristics of the texture databases used in our experiments.

Number	Name	Classes	Samples Per Class	Total Samples	Sample Resolution (Pixels)	Image Format (Monochrome)	Challenges
1	Brodatz	112	9	1008	512 × 512	JPG	Various texture types
2	KTH-TIPS2b	11	4 × 108	4752	200 × 200	BMP	illumination, scale, pose changes
3	OuTeX_TC_00010	24	180	4320	128 × 128	RAS	Rotation changes (0${}^{\circ }$) for training
							and other degrees for test
4	OuTeX_TC_00012	24	200	4800	128 × 128	RAS	Rotation and illumination
							(“Tl84”, “horizon”) changes
5	UMD	25	40	1000	1280 × 960	PNG	Small illumination changes and strong scale,
							rotation, and viewpoint changes

**Table 2:** Classification accuracy (%) of the CRSLBP for different R on the Outex dataset and (SVM, NN) classifier.

	Classification Accuracy (%) Outex (SVM)												Classification Accuracy (%) (NN)			
	**Outex_TC10**				**Outex_TC12**								**Outex_TC10**			
	**Inca**				**T184**				**Horizon**				**Inca**			
	**R = 1**	**R = 2**	**R = 3**	**Average**	**R = 1**	**R = 2**	**R = 3**	**Average**	**R = 1**	**R = 2**	**R = 3**	**Average**	**R = 1**	**R = 2**	**R = 3**	**Average**
LBP classic	96.26	97.10	97.94	97.1	77.83	79.25	78.83	78.64	81.98	82.50	78.69	81.06	96.91	97.38	98.76	97.68
HLBP	92.48	98.40	98.17	96.35	72.52	78.37	74.81	75.23	76.17	79.21	76.02	77.13	93.06	99.23	98.15	96.81
HRLBP	92.69	98.31	98.31	96.43	73.52	78.46	74.58	75.52	75.08	79.42	75.92	76.80	94.60	99.23	98.76	97.53
HLBP+LBP	98.59	99.54	99.70	99.27	84.60	84.50	82.50	83.86	86.65	85.06	82.67	84.79	99.08	99.54	99.69	99.43
HRLBP+RLBP	98.59	99.56	99.72	99.29	85.29	84.60	82.42	84.10	86.83	85.21	82.62	84.88	98.92	100	100	99.64
CPLBP	95.30	96.06	98.01	96.45	76.31	78.04	77.52	77.29	79.94	78.52	79.29	79.25	—	—	—	—
LTP	99.21	99.56	99.42	99.40	85.71	83.92	83.42	84.35	85.92	85.23	82.21	84.45	99.53	99.69	99.84	99.69
CLBP S/M	98.70	99.40	99.49	99.20	85.60	85.62	83.27	84.83	86.98	85.77	82.67	85.14	99.22	99.84	99.69	99.58
CLBP S	96.08	97.13	97.99	97.07	77.92	79.85	78.56	78.78	81.48	82.75	78.35	80.86	95.98	97.99	98.77	97.58
CLBP M	94.40	97.08	98.14	96.54	73.10	76.50	75.00	74.87	77.54	77.58	76.10	77.07	95.37	97.99	98.61	97.32
CLDP	96.23	77.10	71.06	81.46	78.40	59.85	53.40	63.88	81.79	64.81	56.00	67.53	96.23	77.10	71.06	81.46
RLBP	96.32	97.27	97.89	97.16	78.40	78.94	78.40	78.58	81.73	82.60	78.75	81.03	95.98	97.68	98.30	97.32
LBPV	78.75	90.72	93.56	87.67	63.69	78.79	82.96	75.15	70.67	83.42	84.42	79.50	81.01	90.12	92.28	87.80
CRSLBP	99.65	99.84	99.79	99.76	94.23	94.50	93.17	93.97	95.42	94.45	93.87	94.58	99.69	99.84	99.84	99.79
MRELBP	99.90			99.90	87.02			87.02	87.04			87.04	100			100

**Table 3:** Classification accuracy (%) of the CRSLBP for different R on the KTH-TIPS2b, UMD and Brodatz dataset and the SVM and NN classifiers.

(a) Using SVM Classifier												
	**Classification Accuracy (%) Outex (SVM)**											
	**KTH-TIPS2b**				**UMD**				**Brodatz**			
	**R = 1**	**R = 2**	**R = 3**	**Average**	**R = 1**	**R = 2**	**R = 3**	**Average**	**R = 1**	**R = 2**	**R = 3**	**Average**
LBP classic	89.67	88.62	85.94	88.07	97.7	97.6	96.1	97.13	90.77	92.16	91.27	91.40
HLBP	89.41	90.51	89.29	89.74	94.90	95.20	95.30	95.13	84.23	84.33	84.72	84.43
HRLBP	89.44	91.35	89.26	90.02	94.50	94.80	95.00	94.77	83.43	85.02	84.73	84.39
HLBP+LBP	96.00	96.76	96.27	96.34	98.70	99.00	98.60	98.77	93.55	94.35	93.95	93.95
HRLBP+RLBP	96.11	96.55	96.06	96.24	99.00	98.70	98.20	98.63	93.45	94.25	93.75	93.81
LTP	95.73	96.46	95.75	95.98	98.9	98.2	98.3	98.47	93.65	94.84	94.94	94.47
CLBP S/M	94.93	96.14	95.18	95.42	98.8	98.2	98.00	98.33	93.65	94.84	94.94	94.47
CLBP S	89.14	89.27	85.69	80.03	97.60	97.00	96.30	94.20	90.67	93.65	91.47	91.93
CLBP M	85.69	88.15	85.65	86.50	94.70	94.50	93.40	94.20	81.65	84.42	83.63	83.23
CLDP	96.23	77.10	71.06	81.46	97.60	87.10	80.10	88.27	91.07	69.35	56.45	72.29
RLBP	89.58	89.16	85.69	88.14	97.50	97.90	96.60	97.33	91.07	93.06	91.67	91.93
LBPV	78.24	83.12	84.41	81.92	88.40	92.70	92	91.03	64.48	76.49	75.00	71.99
CRSLBP	96.89	96.76	97.19	96.94	98.50	98.40	98.80	98.56	94.15	95.54	95.44	95.04
MRELBP	98.55			98.55	99.60			99.60	97.02			97.02
**(b) Using NN Classifier**												
	**Classification Accuracy (%) Outex (SVM)**											
	**KTH-TIPS2b**				**UMD**				**Brodatz**			
	**R = 1**	**R = 2**	**R = 3**	**Average**	**R = 1**	**R = 2**	**R = 3**	**Average**	**R = 1**	**R = 2**	**R = 3**	**Average**
LBP classic	87.08	84.85	83.59	85.17	96.00	92.67	94.00	94.22	87.42	90.07	90.73	89.40
HLBP	81.77	89.34	89.29	86.8	96.67	95.33	92.66	94.89	87.42	86.09	83.44	85.65
HRLBP	81.62	86.26	89.26	85.71	94.64	94.00	96.67	95.10	84.11	84.11	90.07	86.10
HLBP+LBP	94.25	95.79	96.27	95.44	98.00	99.33	100	99.11	95.37	96.03	94.04	95.15
HRLBP+RLBP	95.23	94.53	96.06	95.23	98.64	99.33	99.33	99.1	93.38	95.37	92.05	93.6
LTP	93.68	94.68	92.70	93.69	98.00	98.66	97.33	98.00	94.04	93.38	94.04	93.82
CLBP S/M	92.15	93.40	91.72	92.42	97.33	98.00	98.00	97.78	92.05	92.71	94.04	92.93
CLBP S	85.97	85.41	81.90	84.43	95.33	96.66	91.33	94.44	89.40	93.38	92.72	91.83
CLBP M	82.60	82.18	82.32	82.37	89.33	92.66	86.66	89.55	84.77	83.44	87.42	85.21
CLDP	96.23	77.10	71.06	81.46	96.66	83.33	75.33	85.11	85.43	60.26	58.94	68.21
RLBP	87.79	83.59	80.78	84.05	94.66	92.66	93.33	93.55	93.38	92.72	90.06	92.05
LBPV	71.39	79.95	72.37	74.57	65.33	84.00	87.33	78.88	59.60	78.15	74.83	70.86
CRSLBP	94.81	95.37	95.65	95.28	99.33	100	98.67	99.33	95.37	98.68	100	98.01
MRELBP	96.49			96.49	100			100	97.35			97.35

**Table 4:** Classification accuracy (%) of CRSLBP compared with MRELBP using a set of R and SVM classifier.

	Outex (TC10,TC12)	KTH-TIPS2b	UMD	Brodatz		
	**Inca**	**T184**	**Horizon**			
MRELBP	99.9%	87.02%	87.04%	98.55%	99.6%	97.02%
CRSLBP	99.88%	94.77%	95.56%	99.22%	99.1%	95.84%

## Data Availability

No new data were created or analyzed in this study. Data sharing is not applicable to this article.

## References

[1] Ojala T., Pietikäinen M., Harwood D. (1996). A comparative study of texture measures with classification based on featured distributions. Pattern Recognit..

[2] Ojala T., Pietikainen M., Maenpaa T. (2002). Multiresolution gray-scale and rotation invariant texture classification with local binary patterns. IEEE Trans. Pattern Anal. Mach. Intell..

[3] Liao S., Law M.W., Chung A.C. (2009). Dominant local binary patterns for texture classification. IEEE Trans. Image Process..

[4] Tan X., Triggs B. (2010). Enhanced local texture feature sets for face recognition under difficult lighting conditions. IEEE Trans. Image Process..

[5] Guo Z., Zhang L., Zhang D. (2010). A completed modeling of local binary pattern operator for texture classification. IEEE Trans. Image Process..

[6] Liu L., Long Y., Fieguth P.W., Lao S., Zhao G. (2014). BRINT: Binary rotation invariant and noise tolerant texture classification. IEEE Trans. Image Process..

[7] Guo Z., Wang X., Zhou J., You J. (2015). Robust texture image representation by scale selective local binary patterns. IEEE Trans. Image Process..

[8] Liu L., Lao S., Fieguth P.W., Guo Y., Wang X., Pietikäinen M. (2016). Median robust extended local binary pattern for texture classification. IEEE Trans. Image Process..

[9] Shakoor M.H., Boostani R. (2018). Radial mean local binary pattern for noisy texture classification. Multimed. Tools Appl..

[10] Kou Q., Cheng D., Zhuang H., Gao R. (2018). Cross-complementary local binary pattern for robust texture classification. IEEE Signal Process. Lett..

[11] Chakraborti T., McCane B., Mills S., Pal U. (2018). Loop descriptor: Local optimal-oriented pattern. IEEE Signal Process. Lett..

[12] Ruichek Y. (2019). Attractive-and-repulsive center-symmetric local binary patterns for texture classification. Eng. Appl. Artif. Intell..

[13] Kas M., Ruichek Y., Messoussi R. (2020). Multi level directional cross binary patterns: New handcrafted descriptor for SVM-based texture classification. Eng. Appl. Artif. Intell..

[14] Xu X., Li Y., Wu Q.J. (2020). A completed local shrinkage pattern for texture classification. Appl. Soft Comput..

[15] Alpaslan N., Hanbay K. (2020). Multi-scale shape index-based local binary patterns for texture classification. IEEE Signal Process. Lett..

[16] Alpaslan N., Hanbay K. (2020). Multi-resolution intrinsic texture geometry-based local binary pattern for texture classification. IEEE Access.

[17] Pan Z., Hu S., Wu X., Wang P. (2021). Adaptive center pixel selection strategy to Local Binary Pattern for texture classification. Expert Syst. Appl..

[18] Shakoor M.H., Boostani R. (2021). Noise robust and rotation invariant texture classification based on local distribution transform. Multimed. Tools Appl..

[19] Ojala T., Maenpaa T., Pietikainen M., Viertola J., Kyllonen J., Huovinen S. Outex-new framework for empirical evaluation of texture analysis algorithms. Proceedings of the 2002 International Conference on Pattern Recognition.

[20] Caputo B., Hayman E., Mallikarjuna P. Class-specific material categorisation. Proceedings of the Tenth IEEE International Conference on Computer Vision (ICCV’05).

[21] Xu Y., Ji H., Fermuller C. A projective invariant for textures. Proceedings of the 2006 IEEE Computer Society Conference on Computer Vision and Pattern Recognition (CVPR’06).

[22] Brodatz P. (1966). Textures: A Photographic Album for Artists and Designers.

[23] Al Saidi I., Rziza M., Debayle J. A New Texture Descriptor: The Homogeneous Local Binary Pattern (HLBP). Proceedings of the International Conference on Image and Signal Processing.

[24] Al Saidi I., Rziza M., Debayle J. A novel texture descriptor: Homogeneous Rotated Local Binary Pattern (HRLBP). Proceedings of the 2020 10th International Symposium on Signal, Image, Video and Communications (ISIVC).

[25] Al Saidi I., Rziza M., Debayle J. (2021). A novel texture descriptor: Circular parts local binary pattern. Image Anal. Stereol..

